# Cohesion and Coalition Formation in the European Parliament: Roll-Call Votes and Twitter Activities

**DOI:** 10.1371/journal.pone.0166586

**Published:** 2016-11-11

**Authors:** Darko Cherepnalkoski, Andreas Karpf, Igor Mozetič, Miha Grčar

**Affiliations:** 1 Department of Knowledge Technologies, Jožef Stefan Institute, Ljubljana, Slovenia; 2 Université Panthéon-Sorbonne / Paris School of Economics, Paris, France; Universidad Nacional de Mar del Plata, ARGENTINA

## Abstract

We study the cohesion within and the coalitions between political groups in the Eighth European Parliament (2014–2019) by analyzing two entirely different aspects of the behavior of the Members of the European Parliament (MEPs) in the policy-making processes. On one hand, we analyze their co-voting patterns and, on the other, their retweeting behavior. We make use of two diverse datasets in the analysis. The first one is the roll-call vote dataset, where cohesion is regarded as the tendency to co-vote within a group, and a coalition is formed when the members of several groups exhibit a high degree of co-voting agreement on a subject. The second dataset comes from Twitter; it captures the retweeting (i.e., endorsing) behavior of the MEPs and implies cohesion (retweets within the same group) and coalitions (retweets between groups) from a completely different perspective. We employ two different methodologies to analyze the cohesion and coalitions. The first one is based on Krippendorff’s Alpha reliability, used to measure the agreement between raters in data-analysis scenarios, and the second one is based on Exponential Random Graph Models, often used in social-network analysis. We give general insights into the cohesion of political groups in the European Parliament, explore whether coalitions are formed in the same way for different policy areas, and examine to what degree the retweeting behavior of MEPs corresponds to their co-voting patterns. A novel and interesting aspect of our work is the relationship between the co-voting and retweeting patterns.

## Introduction

Social-media activities often reflect phenomena that occur in other complex systems. By observing social networks and the content propagated through these networks, we can describe or even predict the interplay between the observed social-media activities and another complex system that is more difficult, if not impossible, to monitor. There are numerous studies reported in the literature that successfully correlate social-media activities to phenomena like election outcomes [1, 2] or stock-price movements [3, 4].

In this paper we study the cohesion and coalitions exhibited by political groups in the Eighth European Parliament (2014–2019). We analyze two entirely different aspects of how the Members of the European Parliament (MEPs) behave in policy-making processes. On one hand, we analyze their co-voting patterns and, on the other, their retweeting (i.e., endorsing) behavior.

We use two diverse datasets in the analysis: the roll-call votes and the Twitter data. A roll-call vote (RCV) is a vote in the parliament in which the names of the MEPs are recorded along with their votes. The RCV data is available as part of the minutes of the parliament’s plenary sessions. From this perspective, cohesion is seen as the tendency to co-vote (i.e., cast the same vote) within a group, and a coalition is formed when members of two or more groups exhibit a high degree of co-voting on a subject. The second dataset comes from Twitter. It captures the retweeting behavior of MEPs and implies cohesion (retweets within the same group) and coalitions (retweets between the groups) from a completely different perspective.

With over 300 million monthly active users and 500 million tweets posted daily, Twitter is one of the most popular social networks. Twitter allows its users to post short messages (tweets) and to follow other users. A user who follows another user is able to read his/her public tweets. Twitter also supports other types of interaction, such as user mentions, replies, and retweets. Of these, retweeting is the most important activity as it is used to share and endorse content created by other users. When a user retweets a tweet, the information about the original author as well as the tweet’s content are preserved, and the tweet is shared with the user’s followers. Typically, users retweet content that they agree with and thus endorse the views expressed by the original tweeter.

We apply two different methodologies to analyze the cohesion and coalitions. The first one is based on Krippendorff’s *Alpha* [5] which measures the agreement among observers, or voters in our case. The second one is based on Exponential Random Graph Models (ERGM) [6]. In contrast to the former, ERGM is a network-based approach and is often used in social-network analyses. Even though these two methodologies come with two different sets of techniques and are based on different assumptions, they provide consistent results.

The main contributions of this paper are as follows:

1. We give general insights into the cohesion of political groups in the Eighth European Parliament, both overall and across different policy areas.
2. We explore whether coalitions are formed in the same way for different policy areas.
3. We explore to what degree the retweeting behavior of MEPs corresponds to their co-voting patterns.
4. We employ two statistically sound methodologies and examine the extent to which the results are sensitive to the choice of methodology. While the results are mostly consistent, we show that the difference are due to the different treatment of non-attending and abstaining MEPs by *Alpha* and ERGM.

**Fig 1:**
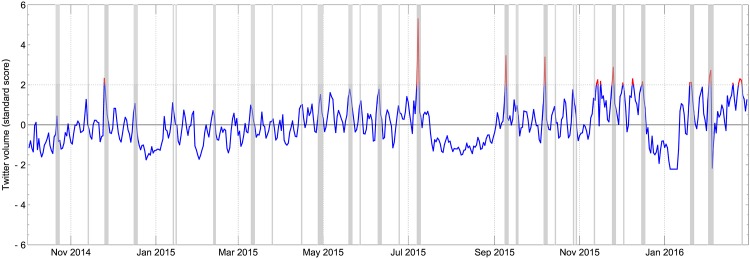
Timeline of Twitter activity of MEPs and roll-call voting sessions. Twitter activity is represented by the total number of tweets posted by all MEPs on a given day. The volume is standardized and the solid blue line represents the standard score. The peaks in Twitter activity that are above two standard deviations are highlighted in red. The shaded regions correspond to days with roll-call voting sessions.

**Fig 2:**
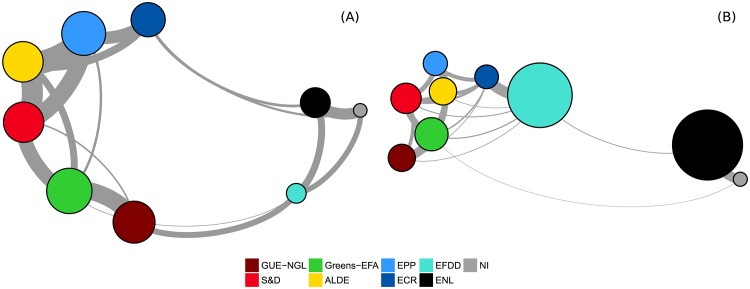
Networks of roll-call votes and retweets. (A) Co-voting agreement within and between political groups. (B) Average retweets within and between political groups.

The most novel and interesting aspect of our work is the relationship between the co-voting and the retweeting patterns. The increased use of Twitter by MEPs on days with a roll-call vote session (see Fig 1) is an indicator that these two processes are related. In addition, the force-based layouts of the co-voting network and the retweet network reveal a very similar structure on the left-to-center side of the political spectrum (see Fig 2). They also show a discrepancy on the far-right side of the spectrum, which calls for a more detailed analysis.

### Related work

In this paper we study and relate two very different aspects of how MEPs behave in policy-making processes. First, we look at their co-voting behavior, and second, we examine their retweeting patterns. Thus, we draw related work from two different fields of science. On one hand, we look at how co-voting behavior is analyzed in the political-science literature and, on the other, we explore how Twitter is used to better understand political and policy-making processes. The latter has been more thoroughly explored in the field of data mining (specifically, text mining and network analysis).

To the best of our knowledge, this is the first paper that studies legislative behavior in the Eighth European Parliament. The legislative behavior of the previous parliaments was thoroughly studied by Hix, Attina, and others [7–12]. These studies found that voting behavior is determined to a large extent—and when viewed over time, increasingly so—by affiliation to a political group, as an organizational reflection of the ideological position. The authors found that the cohesion of political groups in the parliament has increased, while nationality has been less and less of a decisive factor [13]. The literature also reports that a split into political camps on the left and right of the political spectrum has recently replaced the ‘grand coalition’ between the two big blocks of Christian Conservatives (*EPP*) and Social Democrats (*S&D*) as the dominant form of finding majorities in the parliament. The authors conclude that coalitions are to a large extent formed along the left-to-right axis [13].

In this paper we analyze the roll-call vote data published in the minutes of the parliament’s plenary sessions. For a given subject, the data contains the vote of each MEP present at the respective sitting. Roll-call vote data from the European Parliament has already been extensively studied by other authors, most notably by Hix et al. [11, 12, 14]. To be able to study the cohesion and coalitions, authors like Hix, Attina, and Rice [7, 14, 15] defined and employed a variety of agreement measures. The most prominent measure is the Agreement Index proposed by Hix et al. [14]. This measure computes the agreement score from the size of the majority class for a particular vote. The Agreement Index, however, exhibits two drawbacks: (i) it does not account for co-voting by chance, and (ii) without a proper adaptation, it does not accommodate the scenario in which the agreement is to be measured between two different political groups.

We employ two statistically sound methodologies developed in two different fields of science. The first one is based on Krippendorff’s *Alpha* [5]. *Alpha* is a measure of the agreement among observers, coders, or measuring instruments that assign values to items or phenomena. It compares the observed agreement to the agreement expected by chance. *Alpha* is used to measure the inter- and self-annotator agreement of human experts when labeling data, and the performance of classification models in machine learning scenarios [16]. In addition to *Alpha*, we employ Exponential Random Graph Models (ERGM) [6]. In contrast to the former, ERGM is a network-based approach, often used in social-network analyses. ERGM can be employed to investigate how different network statistics (e.g., number of edges and triangles) or external factors (e.g., political group membership) govern the network-formation process.

The second important aspect of our study is related to analyzing the behavior of participants in social networks, specifically Twitter. Twitter is studied by researchers to better understand different political processes, and in some cases to predict their outcomes. Eom et al. [2] consider the number of tweets by a party as a proxy for the collective attention to the party, explore the dynamics of the volume, and show that this quantity contains information about an election’s outcome. Other studies [17] reach similar conclusions. Conover et al. [18] predicted the political alignment of Twitter users in the run-up to the 2010 US elections based on content and network structure. They analyzed the polarization of the retweet and mention networks for the same elections [19]. Borondo et al. [20] analyzed user activity during the Spanish presidential elections. They additionally analyzed the 2012 Catalan elections, focusing on the interplay between the language and the community structure of the network [21]. Most existing research, as Larsson points out [22], focuses on the online behavior of leading political figures during election campaigns.

This paper continues our research on communities that MEPs (and their followers) form on Twitter [23]. The goal of our research was to evaluate the role of Twitter in identifying communities of influence when the actual communities are known. We represent the influence on Twitter by the number of retweets that MEPs “receive”. We construct two networks of influence: (i) core, which consists only of MEPs, and (ii) extended, which also involves their followers. We compare the detected communities in both networks to the groups formed by the political, country, and language membership of MEPs. The results show that the detected communities in the core network closely match the political groups, while the communities in the extended network correspond to the countries of residence. This provides empirical evidence that analyzing retweet networks can reveal real-world relationships and can be used to uncover hidden properties of the networks.

Lazer [24] highlights the importance of network-based approaches in political science in general by arguing that politics is a relational phenomenon at its core. Some researchers have adopted the network-based approach to investigate the structure of legislative work in the US Congress, including committee and sub-committee membership [25], bill co-sponsoring [26], and roll-call votes [27]. More recently, Dal Maso et al. [28] examined the community structure with respect to political coalitions and government structure in the Italian Parliament. Scherpereel et al. [29] examined the constituency, personal, and strategic characteristics of MEPs that influence their tweeting behavior. They suggested that Twitter’s characteristics, like immediacy, interactivity, spontaneity, personality, and informality, are likely to resonate with political parties across Europe. By fitting regression models, the authors find that MEPs from incohesive groups have a greater tendency to retweet.

In contrast to most of these studies, we focus on the Eighth European Parliament, and more importantly, we study and relate two entirely different behavioral aspects, co-voting and retweeting. The goal of this research is to better understand the cohesion and coalition formation processes in the European Parliament by quantifying and comparing the co-voting patterns and social behavior.

## Data

We collected the results of the roll-call votes (RCVs) from the website of the European Parliament [30]. The RCV data is available in the form of XMLs. From the same website, we also obtained the register of active MEPs [31]. This register, even though it is available as XML data, presents a challenge when integrated with the RCV documents. The reason lies in the IDs of MEPs in the registry, which differ from the IDs of the same MEPs in the RCV documents. We used a name-matching technique based on Jaccard distance to establish the missing mappings. Moreover, we linked each MEP to her/his Twitter ID and thus established an explicit link between the MEP and her/his tweeting activities.

The European Parliament consists of 751 members. Within the period of our study, from October 1, 2014 to February 29, 2016, 36 MEPs left the European Parliament and were substituted by 36 new MEPs. This gives a total of 787 MEPs that participated in the work of the parliament during this period. In total, 79% of the MEPs have Twitter accounts and 67% have retweeted or been retweeted by another MEP. The distribution of MEPs by political groups, with their Twitter accounts, and retweeting activity is given in Table 1.

**Table 1:** Distribution of Members of the European Parliament (MEPs) by political groups. There is the number of MEPs in each group, the number of MEPs with Twitter accounts, and the number of MEPs in the retweet network. The numbers are cumulative for the period from October 1, 2014 to February 29, 2016.

Political group	Number of MEPs	Twitter accounts	Nodes in the retweet network
GUE-NGL	61	43	41
S&D	193	162	135
Greens-EFA	51	48	44
ALDE	77	62	54
EPP	223	166	141
ECR	82	64	48
EFDD	45	39	34
ENL	38	29	27
NI	17	9	6
Total	787	622	530

Political groups are ordered by their seating positions in the European Parliament from the left to the right wing:

- *GUE-NGL:* The European United Left-Nordic Green Left unite radical left wing, socialist and communist parties. They support a social union with stronger protection of workers and a higher degree of wealth redistribution.
- *S&D:* The Socialists & Democrats consist of social democratic, socialist and labour parties. They have a balanced stance between workers protection and market economy, and are integration friendly.
- *Greens-EFA:* The Group of the Greens and European Free Alliance is the political home to the Green and regionalist/separatist political parties (EFA). The group is favorable to European integration, and strives for a more ecological and social union.
- *ALDE:* The Alliance of Liberals and Democrats for Europe summons liberal political groups. They are cooperation and integration friendly, with a stance against overregulation and bureaucratization.
- *EPP:* The European People’s Party unites christian democrats, conservatives and center-right parties. They are considered integration-friendly and strong supporters of the European Constitution.
- *ECR:* European Conservatives and Reformists provide a political home to conservative and Eurosceptic parties.
- *EFDD:* The Europe of Freedom and Direct Democracy unites extreme right-wing and Eurosceptic parties.
- *ENL:* The Europe of Nations and Freedom is the smallest group, with extreme right-wing members.
- *NI:* There are also some non-aligned (Non-Inscrits NI) MEPs, partly split-offs from national lists, or members of other minority extreme-right wing political movements.

A detailed description of all the political groups in the European Parliament is in S1 Appendix.

In the time frame of this study, the MEPs voted in 2535 roll-call votes. Of these, 2358 RCVs contain information about the policy area (referred to as *subject* in the RCV metadata) they are part of. The distribution of RCVs by policy area is given in Table 2.

**Table 2:** Distribution of roll-call votes (RCVs) by policy areas. The time period is from October 1, 2014 to February 29, 2016.

Policy area	RCVs
Area of freedom, security and justice	116
Community policies	568
Economic and monetary system	105
Economic, social and territorial cohesion	314
European citizenship	92
External relations of the Union	512
Internal market	232
State and evolution of the Union	419
Unclassified	177
Total	2535

From the data collected, a matrix of 787 MEPs by 2535 RCVs is formed. Each entry is either *yes* or *no* (depending how the MEP voted in the RCV), or missing (when the MEP abstained or did not attend the RCV session). The matrix is used to compute the Krippendorff’s *Alpha* co-voting agreement. For the ERGM analysis, a network is formed for each RCV. The nodes are all the 787 MEPs and there is an edge between two MEPs if they cast the same vote (*yes* or *no*). MEPs that abstained, or did not attend the RCV session, are disconnected nodes.

The retweet network of the parliament consists of 530 nodes corresponding to the MEPs that are active on Twitter, and 4723 edges. An undirected edge between two MEPs is formed when one of them retweeted the other. The weight of the edge represents the number of times these MEPs have retweeted each other. The total weight of edges in the network, corresponding to the total number of retweets between the MEPs, is 26133.

## Methods

In this section we present the methods to quantify cohesion and coalitions from the roll-call votes and Twitter activities.

### Co-voting measured by agreement

We first show how the co-voting behaviour of MEPs can be quantified by a measure of the agreement between them. We treat individual RCVs as observations, and MEPs as independent observers or raters. When they cast the same vote, there is a high level of agreement, and when they vote differently, there is a high level of disagreement. We define cohesion as the level of agreement within a political group, a coalition as a voting agreement between political groups, and opposition as a disagreement between different groups.

There are many well-known measures of agreement in the literature. We selected Krippendorff’s Alpha-reliability (*Alpha*) [5], which is a generalization of several specialized measures. It works for any number of observers, and is applicable to different variable types and metrics (e.g., nominal, ordered, interval, etc.). In general, *Alpha* is defined as follows:

$$\begin{array}{c} Alpha=1-\frac{D_{o}}{D_{e}}\;, \end{array}$$

where *D*_*o*_ is the actual disagreement between observers (MEPs), and *D*_*e*_ is disagreement expected by chance. When observers agree perfectly, *Alpha* = 1, when the agreement equals the agreement by chance, *Alpha* = 0, and when the observers disagree systematically, *Alpha* < 0.

The two disagreement measures are defined as follows:

$$\begin{array}{c} D_{o}=\frac{1}{N}\sum_{c,c^{\prime }}N\left(c,c^{\prime }\right)\cdot \delta^{2}\left(c,c^{\prime }\right)\;, \end{array}$$

$$\begin{array}{c} D_{e}=\frac{1}{N(N-1)}\sum_{c,c^{\prime }}N\left(c\right)\cdot N\left(c^{\prime }\right)\cdot \delta^{2}\left(c,c^{\prime }\right)\;. \end{array}$$

The arguments *N*, *N*(*c*, *c*′), *N*(*c*), and *N*(*c*′) are defined below and refer to the values in the coincidence matrix that is constructed from the RCVs data. In roll-call votes, *c* (and *c*′) is a nominal variable with two possible values: *yes* and *no*. *δ*(*c*, *c*′) is a difference function between the values of *c* and *c*′, defined as:

$$\begin{array}{c} \delta \left(c,c^{\prime }\right)=\{\begin{array}{cc} 0 & \;\text{iff}\;c=c^{\prime }\\ 1 & \;\text{iff}\;c\neq c^{\prime } \end{array}\;\;\;\;c,c^{\prime }\in \left\{yes,no\right\}\;\;. \end{array}$$

The RCVs data has the form of a reliability data matrix:

$$\begin{array}{c} \begin{array}{cccccccc} \text{Roll-call}\;\text{votes} & & 1 & 2 & \cdots & u & \cdots & n\\ & 1 & c_{11} & c_{12} & \cdots & c_{1u} & \cdots & c_{1n}\\ \text{Individual} & . & . & . & \cdots & . & \cdots & .\\ \text{MEPs}\;\text{voting} & i & c_{i1} & c_{i2} & \cdots & c_{iu} & \cdots & c_{in}\\ & . & . & . & \cdots & . & \cdots & .\\ & m & c_{m1} & c_{m2} & \cdots & c_{mu} & \cdots & c_{mn}\\ \text{No.}\;\text{of}\;\text{MEPs}\;\text{voting} & & m_{1} & m_{2} & \cdots & m_{u} & \cdots & m_{n} \end{array} \end{array}$$

where *n* is the number of RCVs, *m* is the number of MEPs, *m*_*u*_ is the number of votes cast in the voting *u*, and *c*_*iu*_ is the actual vote of an MEP *i* in voting *u* (*yes* or *no*).

A coincidence matrix is constructed from the reliability data matrix, and is in general a *k*-by-*k* square matrix, where *k* is the number of possible values of *c*. In our case, where only *yes/no* votes are relevant, the coincidence matrix is a 2-by-2 matrix of the following form:

$$\begin{array}{c} \begin{array}{cccc} & & c^{\prime } & \sum \\ & . & . & .\\ c & . & N(c,c^{\prime }) & N(c)\\ \sum & . & N(c^{\prime }) & N \end{array} \end{array}$$

A cell *N*(*c*, *c*′) accounts for all coincidences from all pairs of MEPs in all RCVs where one MEP has voted *c* and the other *c*′. *N*(*c*) and *N*(*c*′) are the totals for each vote outcome, and *N* is the grand total. The coincidences *N*(*c*, *c*′) are computed as:

$$\begin{array}{c} N\left(c,c^{\prime }\right)=\sum_{u}\frac{N_{u}\left(c,c^{\prime }\right)}{m_{u}-1}\;\;\;\;c,c^{\prime }\in \left\{yes,no\right\} \end{array}$$

where *N*_*u*_(*c*, *c*′) is the number of (*c*, *c*′) pairs in vote *u*, and *m*_*u*_ is the number of MEPs that voted in *u*. When computing *N*_*u*_(*c*, *c*′), each pair of votes is considered twice, once as a (*c*, *c*′) pair, and once as a (*c*′, *c*) pair. The coincidence matrix is therefore symmetrical around the diagonal, and the diagonal contains all the equal votes.

The *Alpha* agreement is used to measure the agreement between two MEPs or within a group of MEPs. When applied to a political group, *Alpha* corresponds to the cohesion of the group. The closer *Alpha* is to 1, the higher the agreement of the MEPs in the group, and hence the higher the cohesion of the group.

We propose a modified version of *Alpha* to measure the agreement between two different groups, *A* and *B*. In the case of a voting agreement between political groups, high *Alpha* is interpreted as a coalition between the groups, whereas negative *Alpha* indicates political opposition.

Suppose *A* and *B* are disjoint subsets of all the MEPs, *A*, *B* ⊂ {1, …, *m*}, *A* ∩ *B* = {}. The respective number of votes cast by both group members in vote *u* is *m*_*u*_(*A*) and *m*_*u*_(*B*). The coincidences are then computed as:

$$\begin{array}{c} N\left(c,c^{\prime }\right)=\sum_{u}\frac{N_{u}\left(c,c^{\prime }\right)\cdot \left(m_{u}\left(A\right)+m_{u}\left(B\right)\right)}{2\cdot m_{u}\left(A\right)\cdot m_{u}\left(B\right)}\;\;\;\;c,c^{\prime }\in \left\{yes,no\right\} \end{array}$$

where the (*c*, *c*′) pairs come from different groups, *A* and *B*. The total number of such pairs in vote *u* is *m*_*u*_(*A*) ⋅ *m*_*u*_(*B*). The actual number *N*_*u*_(*c*, *c*′) of the pairs is multiplied by $\frac{m_{u}\left(A\right)+m_{u}\left(B\right)}{2\cdot m_{u}\left(A\right)\cdot m_{u}\left(B\right)}$ so that the total contribution of vote *u* to the coincidence matrix is *m*_*u*_(*A*) + *m*_*u*_(*B*).

### A network-based measure of co-voting

In this section we describe a network-based approach to analyzing the co-voting behavior of MEPs. For each roll-call vote we form a network, where the nodes in the network are MEPs, and an undirected edge between two MEPs is formed when they cast the same vote.

We are interested in the factors that determine the cohesion within political groups and coalition formation between political groups. Furthermore, we investigate to what extent communication in a different social context, i.e., the retweeting behavior of MEPs, can explain the co-voting of MEPs. For this purpose we apply an Exponential Random Graph Model [6] to individual roll-call vote networks, and aggregate the results by means of the meta-analysis.

#### Exponential Random Graph Model

Exponential Random Graph Models (ERGM) allow us to investigate the factors relevant for the network-formation process. Network metrics, as described in the abundant literature, serve to gain information about the structural properties of the observed network. A model investigating the processes driving the network formation, however, has to take into account that there can be a multitude of alternative networks. If we are interested in the parameters influencing the network formation we have to consider all possible networks and measure their similarity to the originally observed network. The family of ERGMs builds upon this idea.

Assume a random graph *Y*, in the form of a binary adjacency matrix, made up of a set of *n* nodes and *e* edges {*Y*_*ij*_ : *i* = 1, …, *n*; *j* = 1, …, *n*} where, similar to a binary choice model, *Y*_*ij*_ = 1 if the nodes (*i*, *j*) are connected and *Y*_*ij*_ = 0 if not. Since network data is by definition relational and thus violates assumptions of independence, classical binary choice models, like logistic regression, cannot be applied in this context. Within an ERGM, the probability for a given network is modelled by

$$\begin{array}{c} P\left(Y=y|\theta \right)=\frac{\exp(\theta^{T}\cdot g\left(y\right))}{c(\theta )}\;\;, \end{array}$$ (1)

where *θ* is the vector of parameters and *g*(*y*) is the vector of network statistics (counts of network substructures), which are a function of the adjacency matrix *y*. *c*(*θ*) = ∑exp(*θ*^*T*^ ⋅ *g*(*y*)) is a normalization constant corresponding to the sample of all possible networks, which ensures a proper probability distribution. Evaluating the above expression allows us to make assertions if and how specific nodal attributes influence the network formation process. These nodal attributes can be endogenous (dyad-dependent parameters) to the network, like the in- and out-degrees of a node, or exogenous (dyad-independent parameters), as the party affiliation, or the country of origin in our case.

#### Interpretation of the coefficients

An alternative formulation of the ERGM provides the interpretation of the coefficients. We introduce the change statistic, which is defined as the change in the network statistics when an edge between nodes *i* and *j* is added or not. If $g(y_{ij}^{+})$ and $g(y_{ij}^{-})$ denote the vectors of counts of network substructures when the edge is added or not, the change statistics is defined as follows:

$$\begin{array}{c} \Delta y_{ij}=g\left(y_{ij}^{+}\right)-g\left(y_{ij}^{-}\right)\;\;. \end{array}$$

With this at hand it can be shown that the distribution of the variable *Y*_*ij*_, conditional on the rest of the graph $Y_{ij}^{c}$, corresponds to:

$$\begin{array}{c} \log(\frac{p(Y_{ij}=1|Y_{ij}^{c}=y_{ij}^{c})}{p(Y_{ij}=0|Y_{ij}^{c}=y_{ij}^{c})})=logit\left(p\left(Y_{ij}=1|Y_{ij}^{c}=y_{ij}^{c}\right)\right)=\theta^{T}\Delta y_{ij}\;\;. \end{array}$$

This implies on the one hand that the probability depends on $y_{ij}^{c}$ via the change statistic Δ*y*_*ij*_, and on the other hand, that each coefficient within the vector *θ* represents an increase in the conditional log-odds (*logit*) of the graph when the corresponding element in the vector *g*(*y*) increases by one. The need to condition the probability on the rest of the network can be illustrated by a simple example. The addition (removal) of a single edge alters the network statistics. If a network has only edges (*j*, *k*) and (*i*, *k*), the creation of an edge (*i*, *j*) would not only add an additional edge but would also alter the count for other network substructures included in the model. In this example, the creation of the edge (*i*, *j*) also increases the number of triangles by one. The coefficients are transformed into probabilities with the logistic function:

$$\begin{array}{c} p=\frac{1}{1+exp(-\theta )}\;\;. \end{array}$$

For example, in the context of roll-call votes, the probability that an additional co-voting edge is formed between two nodes (MEPs) of the same political group is computed with that equation. In this context, the nodematch (nodemix) coefficients of the ERGM (described in detail bellow) therefore refer to the degree of homophilous (heterophilous) matching of MEPs with regard to their political affiliation, or, expressed differently, the propensity of MEPs to co-vote with other MEPs of their respective political group or another group.

A positive coefficient reflects an increased chance that an edge between two nodes with respective properties, like group affiliation, given all other parameters unchanged, is formed. Or, put differently, a positive coefficient implies that the probability of observing a network with a higher number of corresponding pairs relative to the hypothetical baseline network, is higher than to observe the baseline network itself [32]. For an intuitive interpretation, log-odds value of 0 corresponds to the even chance probability of 0.5. Log-odds of +1 correspond to an increase of probability by 0.23, whereas log-odds of −1 correspond to a decrease of probability by 0.23.

#### Sampling and estimation

The computational challenges of estimating ERGMs is to a large degree due to the estimation of the normalizing constant. The number of possible networks is already extremely large for very small networks and the computation is simply not feasible. Therefore, an appropriate sample has to be found, ideally covering the most probable areas of the probability distribution. For this we make use of a method from the Markov Chain Monte Carlo family, namely the Metropolis-Hastings algorithm.

The idea behind this algorithm is to generate and sample highly weighted random networks departing from the observed network. The Metropolis-Hastings algorithm is an iterative algorithm which samples from the space of possible networks by randomly adding or removing edges from the starting network conditional on its density. If the likelihood, in the ERGM context also denoted as weights, of the newly generated network is higher than that of the departure network it is retained, otherwise it is discarded. In the former case, the algorithm starts anew from the newly generated network. Otherwise departure network is used again. Repeating this procedure sufficiently often and summing the weights associated to the stored (sampled) networks allows to compute an approximation of the denominator in Eq (1) (normalizing constant).

The algorithm starts sampling from the originally observed network *G*. The optimization of the coefficients is done simultaneously, equivalently with the Metropolis-Hastings algorithm. At the beginning starting values have to be supplied. For the study at hand we used the “ergm” library from the statistical R software package [6] implementing the Gibbs-Sampling algorithm [33] which is a special case of the Metropolis-Hastings algorithm outlined.

#### Specification of the ERGM

In order to answer our question of the importance of the factors which drive the network formation process in the roll-call co-voting network, the ERGM is specified with the following parameters:

1. *nodematch country:* This parameter adds one network statistic to the model, i.e., the number of edges (*i*, *j*) where *country*_*i*_ = *country*_*j*_. The coefficient indicates the homophilious mixing behavior of MEPs with respect to their country of origin. In other words, this coefficient indicates how relevant nationality is in the formation of edges in the co-voting network.
2. *nodematch national party:* This parameter adds one network statistic to the model: the number of edges (*i*, *j*) with *national*.*party*_*i*_ = *national*.*party*_*j*_. The coefficient indicates the homophilious mixing behavior of the MEPs with regard to their party affiliation at the national level. In the context of this study, this coefficient can be interpreted as an indicator for within-party cohesion at the national level.
3. *nodemix EP group:* This parameter adds one network statistic for each pair of European political groups. These coefficients shed light on the degree of coalitions between different groups as well as the within group cohesion. Given that there are nine groups in the European Parliament, this coefficient adds in total 81 statistics to the model.
4. *edge covariate Twitter:* This parameter corresponds to a square matrix with the dimension of the adjacency matrix of the network, which corresponds to the number of mutual retweets between the MEPs. It provides an insight about the extent to which communication in one social context (Twitter), can explain cooperation in another social context (co-voting in RCVs).

#### Meta-analysis

An ERGM as specified above is estimated for each of the 2535 roll-call votes. Each roll-call vote is thereby interpreted as a binary network and as an independent study. It is assumed that a priori each MEP could possibly form an edge with each other MEP in the context of a roll-call vote. Assumptions over the presence or absence of individual MEPs in a voting session are not made. In other words the dimensions of the adjacency matrix (the node set), and therefore the distribution from which new networks are drawn, is kept constant over all RCVs and therefore for every ERGM. The ERGM results therefore implicitly generalize to the case where potentially all MEPs are present and could be voting. Not voting is incorporated implicitly by the disconnectedness of a node.

The coefficients of the 2535 roll-call vote studies are aggregated by means of a meta-analysis approach proposed by Lubbers [34] and Snijders et al. [35]. We are interested in average effect sizes of different matching patterns over different topics and overall. Considering the number of RCVs, it seems straightforward to interpret the different RCV networks as multiplex networks and collapse them into one weighted network, which could then be analysed by means of a valued ERGM [36]. There are, however, two reasons why we chose the meta-analysis approach instead. First, aggregating the RCV data results into an extremely dense network, leading to severe convergence (degeneracy) problems for the ERGM. Second, the RCV data contains information about the different policy areas the individual votes were about. Since we are interested in how the coalition formation in the European Parliament differs over different areas, a method is needed that allows for an ex-post analysis of the corresponding results. We therefore opted for the meta-analysis approach by Lubbers and Snijders et al. This approach allows us to summarize the results by decomposing the coefficients into average effects and (class) subject-specific deviations. The different ERGM runs for each RCV are thereby regarded as different studies with identical samples that are combined to obtain a general overview of effect sizes. The meta-regression model is defined as:

$$\begin{array}{c} \theta_{m}=\mu_{\theta }+E_{m}\;\;. \end{array}$$

Here *θ*_*m*_ is a parameter estimate for class *m*, and *μ*_*θ*_ is the average coefficient. *E*_*m*_ denotes the normally distributed deviation of the class *m* with a mean of 0 and a variance of *σ*^2^. *E*_*m*_ is the estimation error of the parameter value *θ*_*m*_ from the ERGM. The meta-analysis model is fitted by an iterated, weighted, least-squares model in which the observations are weighted by the inverse of their variances. For the overall nodematch between political groups, we weighted the coefficients by group sizes. The results from the meta analysis can be interpreted as if they stemmed from an individual ERGM run.

In our study, the meta-analysis was performed using the RSiena library [37], which implements the method proposed by Lubbers and Snijders et al. [34, 35].

### Measuring cohesion and coalitions on Twitter

The retweeting behavior of MEPs is captured by their retweet network. Each MEP active on Twitter is a node in this network. An edge in the network between two MEPs exists when one MEP retweeted the other. The weight of the edge is the number of retweets between the two MEPs. The resulting retweet network is an undirected, weighted network.

We measure the cohesion of a political group *A* as the average retweets, i.e., the ratio of the number of retweets between the MEPs in the group *retweets*(*A*) to the number of MEPs in the group |*A*|. The higher the ratio, the more each MEP (on average) retweets the MEPs from the same political group, hence, the higher the cohesion of the political group. The definition of the *average retweeets* ($\bar{RT}$) of a group *A* is:

$$\begin{array}{c} \bar{RT}\left(A\right)=\frac{retweets(A)}{\left|A\right|}\;\;. \end{array}$$

This measure of cohesion captures the aggregate retweeting behavior of the group. If we consider retweets as endorsements, a larger number of retweets within the group is an indicator of agreement between the MEPs in the group. It does not take into account the patterns of retweeting within the group, thus ignoring the social sub-structure of the group. This is a potentially interesting direction and we leave it for future work.

We employ an analogous measure for the strength of coalitions in the retweet network. The coalition strength between two groups *A* and *B* is the ratio of the number of retweets from one group to the other (but not within groups) *retweets*(*A*, *B*) to the total number of MEPs in both groups, |*A*| + |*B*|. The definition of the *average retweeets* ($\bar{RT}$) between groups *A* and *B* is:

$$\begin{array}{c} \bar{RT}\left(A,B\right)=\frac{retweets(A,B)}{\left|A|+|B\right|}\;\;. \end{array}$$

## Results

### Cohesion of political groups

In this section we first report on the level of cohesion of the European Parliament’s groups by analyzing the co-voting through the agreement and ERGM measures. Next, we explore two important policy areas, namely *Economic and monetary system* and *State and evolution of the Union*. Finally, we analyze the cohesion of the European Parliament’s groups on Twitter.

#### Co-voting cohesion

Existing research by Hix et al. [11, 12, 14] shows that the cohesion of the European political groups has been rising since the 1990s, and the level of cohesion remained high even after the EU’s enlargement in 2004, when the number of MEPs increased from 626 to 732.

**Fig 3:**
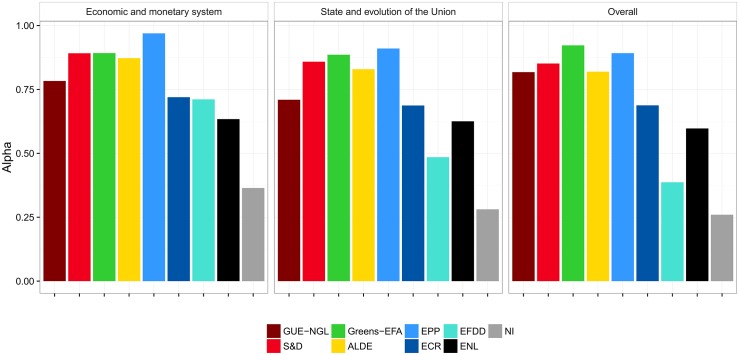
Cohesion of political groups in terms of RCVs as measured by Krippendorff’s *Alpha*. There are two selected policy areas (the left-hand panels), and overall cohesion across all policy areas (the right-hand panel). The *Alpha*-agreement of 1 indicates perfect co-voting agreement, and 0 indicates co-voting by chance. The overall average *Alpha* across all nine political groups is 0.7.

We measure the co-voting cohesion of the political groups in the Eighth European Parliament using Krippendorff’s *Alpha*—the results are shown in Fig 3 (panel *Overall*). The *Greens-EFA* have the highest cohesion of all the groups. This finding is in line with an analysis of previous compositions of the Fifth and Sixth European Parliaments by Hix and Noury [12], and the Seventh by VoteWatch [38]. They are closely followed by the *S&D* and *EPP*. Hix and Noury reported on the high cohesion of *S&D* in the Fifth and Sixth European Parliaments, and we also observe this in the current composition. They also reported a slightly less cohesive *EPP-ED*. This group split in 2009 into *EPP* and *ECR*. VoteWatch reports *EPP* to have cohesion on a par with *Greens-EFA* and *S&D* in the Seventh European Parliament. The cohesion level we observe in the current European Parliament is also similar to the level of *Greens-EFA* and *S&D*.

The catch-all group of the non-aligned (*NI*) comes out as the group with the lowest cohesion. In addition, among the least cohesive groups in the European Parliament are the Eurosceptics *EFDD*, which include the British *UKIP* led by Nigel Farage, and the *ENL* whose largest party are the French *National Front*, led by Marine Le Pen. Similarly, Hix and Noury found that the least cohesive groups in the Seventh European Parliament are the nationalists and Eurosceptics. The Eurosceptic *IND/DEM*, which participated in the Sixth European Parliament, transformed into the current *EFDD*, while the nationalistic *UEN* was dissolved in 2009.

**Fig 4:**
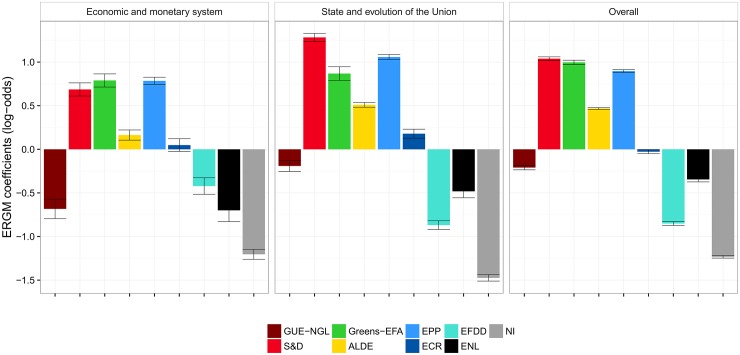
Cohesion of political groups in terms of RCVs as measured by ERGM. There are two selected policy areas (the left-hand panels), and overall cohesion across all policy areas (the right-hand panel). The coefficients of 0 indicate baseline, i.e., an even chance of the same votes within a group. Log-odds of +1 (−1) correspond to an increase (decrease) of probability for 0.23 of co-voting together.

We also measure the voting cohesion of the European Parliament groups using an ERGM, a network-based method—the results are shown in Fig 4 (panel *Overall*). The cohesion results obtained with ERGM are comparable to the results based on agreement. In this context, the parameters estimated by the ERGM refer to the matching of MEPs who belong to the same political group (one parameter per group). The parameters measure the homophilous matching between MEPs who have the same political affiliation. A positive value for the estimated parameter indicates that the co-voting of MEPs from that group is greater than what is expected by chance, where the expected number of co-voting links by chance in a group is taken to be uniformly random. A negative value indicates that there are fewer co-voting links within a group than expected by chance.

Even though *Alpha* and ERGM compute scores relative to what is expected by chance, they refer to different interpretations of chance. *Alpha*’s concept of chance is based on the number of expected pair-wise co-votes between MEPs belonging to a group, knowing the votes of these MEPs on all RCVs. ERGM’s concept of chance is based on the number of expected pair-wise co-votes between MEPs belonging to a group on a given RCV, knowing the network-related properties of the co-voting network on that particular RCV. The main difference between *Alpha* and ERGM, though, is the treatment of non-voting and abstained MEPs. *Alpha* considers only the yes/no votes, and consequently, agreements by the voting MEPs of the same groups are considerably higher than co-voting by chance. ERGM, on the other hand, always considers all MEPs, and non-voting and abstained MEPs are treated as disconnected nodes. The level of co-voting by chance is therefore considerably lower, since there is often a large fraction of MEPs that do not attand or abstain.

As with *Alpha*, *Greens-EFA*, *S&D*, and *EPP* exhibit the highest cohesion, even though their ranking is permuted when compared to the ranking obtained with *Alpha*. At the other end of the scale, we observe the same situation as with *Alpha*. The non-aligned members *NI* have the lowest cohesion, followed by *EFDD* and *ENL*.

The only place where the two methods disagree is the level of cohesion of *GUE-NGL*. The *Alpha* attributes *GUE-NGL* a rather high level of cohesion, on a par with *ALDE*, whereas the ERGM attributes them a much lower cohesion. The reason for this difference is the relatively high abstention rate of *GUE-NGL*. Whereas the overall fraction of non-attending and abstaining MEPs across all RCVs and all political groups is 25%, the *GUE-NGL* abstention rate is 34%. This is reflected in an above average cohesion by *Alpha* where only yes/no votes are considered, and in a relatively lower, below average cohesion by ERGM. In the later case, the non-attendance is interpreted as a non-cohesive voting of a political groups as a whole.

In addition to the overall cohesion, we also focus on two selected policy areas. A detailed description of all the areas is in S1 Appendix. The cohesion of the political groups related to these two policy areas is shown in the first two panels in Fig 3 (*Alpha*) and Fig 4 (ERGM).

The most important observation is that the level of cohesion of the political groups is very stable across different policy areas. These results are corroborated by both methodologies. Similar to the overall cohesion, the most cohesive political groups are the *S&D*, *Greens-EFA*, and *EPP*. The least cohesive group is the *NI*, followed by the *ENL* and *EFDD*. The two methodologies agree on the level of cohesion for all the political groups, except for *GUE-NGL*, due to a lower attendance rate.

#### Cohesion on Twitter

**Fig 5:**
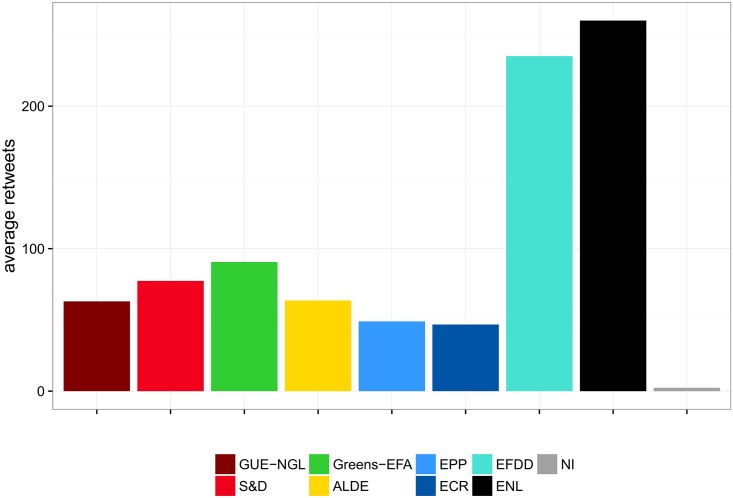
Cohesion of political groups as estimated by retweeting within groups. The average number of retweets within the nine groups is 99.

We determine the cohesion of political groups on Twitter by using the average number of retweets between MEPs within the same group. The results are shown in Fig 5. The right-wing *ENL* and *EFDD* come out as the most cohesive groups, while all the other groups have a far lower average number of retweets. MEPs from *ENL* and *EFDD* post by far the largest number of retweets (over 240), and at the same time over 94% of their retweets are directed to MEPs from the same group. Moreover, these two groups stand out in the way the retweets are distributed within the group. A large portion of the retweets of *EFDD* (1755) go to Nigel Farage, the leader of the group. Likewise, a very large portion of retweets of *ENL* (2324) go to Marine Le Pen, the leader of the group. Farage and Le Pen are by far the two most retweeted MEPs, with the third one having only 666 retweets.

### Coalitions in the European Parliament

Coalition formation in the European Parliament is largely determined by ideological positions, reflected in the degree of cooperation of parties at the national and European levels. The observation of ideological inclinations in the coalition formation within the European Parliament was already made by other authors [12] and is confirmed in this study. The basic patterns of coalition formation in the European Parliament can already be seen in the co-voting network in Fig 2A. It is remarkable that the degree of attachment between the political groups, which indicates the degree of cooperation in the European Parliament, nearly exactly corresponds to the left-to-right seating order.

The liberal *ALDE* seems to have an intermediator role between the left and right parts of the spectrum in the parliament. Between the extreme (*GUE-NGL*) and center left (*S&D*) groups, this function seems to be occupied by *Greens-EFA*. The non-aligned members *NI*, as well as the Eurosceptic *EFFD* and *ENL*, seem to alternately tip the balance on both poles of the political spectrum. Being ideologically more inclined to vote with other conservative and right-wing groups (*EPP*, *ECR*), they sometimes also cooperate with the extreme left-wing group (*GUE-NGL*) with which they share their Euroscepticism as a common denominator.

#### Co-voting coalitions

**Fig 6:**
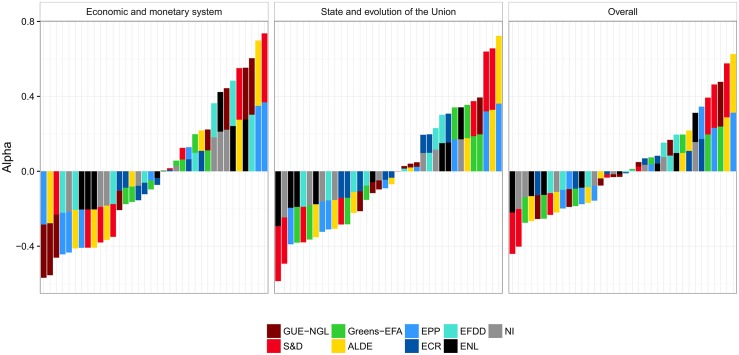
Coalitions between political groups in terms of RCVs as measured by Krippendorff’s *Alpha*. For nine groups, there are 36 pairs for which we measure their (dis)agreement. Positive values of *Alpha* indicate co-voting agreement, negative values correspond to systematic disagreement, while *Alpha* = 0 indicates co-voting by chance. The average *Alpha* across all 36 pairs is 0.02, close to co-voting by chance. Note, however, that *Alpha* considers just yes/no votes, and ignores abstentions.

**Fig 7:**
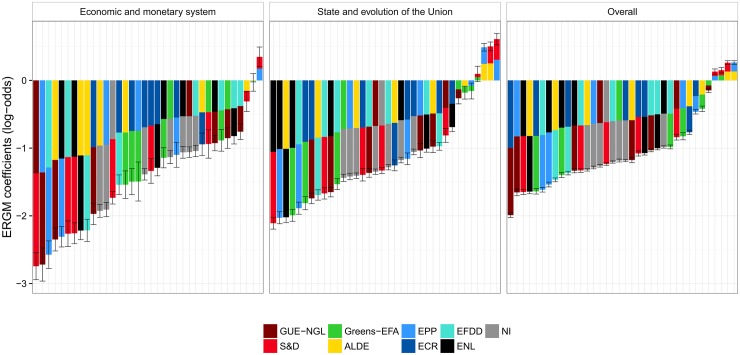
Coalitions between political groups in terms of RCVs as measured by ERGM. There are 36 possible pairwise coalitions of the nine groups. The coefficients of 0 indicate baseline, i.e., an even chance (0.5) of the two groups to co-vote together. For most group pairs, the probability to co-vote is lower than the chance: log-odds of −1 correspond to the probability of 0.27, log-odds of −2 to the probability of 0.12, and log-odds of −3 to the probability of 0.05. Note that ERGM does take abstentions into account, and therefore the baseline of co-voting by chance is considerably higher than for *Alpha*.

Figs 6 and 7 give a more detailed understanding of the coalition formation in the European Parliament. Fig 6 displays the degree of agreement or cooperation between political groups measured by Krippendorff’s *Alpha*, whereas Fig 7 is based on the result from the ERGM. We first focus on the overall results displayed in the right-hand plots of Figs 6 and 7.

The strongest degrees of cooperation are observed, with both methods, between the two major parties (*EPP* and *S&D*) on the one hand, and the liberal *ALDE* on the other. Furthermore, we see a strong propensity for *Greens-EFA* to vote with the Social Democrats (5th strongest coalition by *Alpha*, and 3rd by ERGM) and the *GUE-NGL* (3rd strongest coalition by *Alpha*, and 5th by ERGM). These results underline the role of *ALDE* and *Greens-EFA* as intermediaries for the larger groups to achieve a majority. Although the two largest groups together have 405 seats and thus significantly more than the 376 votes needed for a simple majority, the degree of cooperation between the two major groups is ranked only as the fourth strongest by both methods. This suggests that these two political groups find it easier to negotiate deals with smaller counterparts than with the other large group. This observation was also made by Hix et al. [13], who noted that alignments on the left and right of the political spectrum have in recent years replaced the “Grand Coalition” between the two large blocks of Christian Conservatives (*EPP*) and Social Democrats (*S&D*) as the dominant form of finding majorities in the parliament.

#### Coalitions within individual policy areas

Next, we focus on the coalition formation within the two selected policy areas. The area *State and Evolution of the Union* is dominated by cooperation between the two major groups, *S&D* and *EPP*, as well as *ALDE*. We also observe a high degree of cooperation between groups that are generally regarded as integration friendly, like *Greens-EFA* and *GUE-NGL*. We see, particularly in Fig 6, a relatively high degree of cooperation between groups considered as Eurosceptic, like *ECR*, *EFFD*, *ENL*, and the group of non-aligned members.

The dichotomy between supporters and opponents of European integration is even more pronounced within the policy area *Economic and Monetary System*. In fact, we are taking a closer look specifically at these two areas as they are, at the same time, both contentious and important. Both methods rank the cooperation between *S&D* and *EPP* on the one hand, and *ALDE* on the other, as the strongest.

We also observe a certain degree of unanimity among the Eurosceptic and right-wing groups (*EFDD*, *ENL*, and *NI*) in this policy area. This seems plausible, as these groups were (especially in the aftermath of the global financial crisis and the subsequent European debt crisis) in fierce opposition to further payments to financially troubled member states. However, we also observe a number of strong coalitions that might, at first glance, seem unusual, specifically involving the left-wing group *GUE-NGL* on the one hand, and the right-wing *EFDD*, *ENL*, and *NI* on the other. These links also show up in the network plot in Fig 2A. This might be attributable to a certain degree of Euroscepticism on both sides: rooted in criticism of capitalism on the left, and at least partly a *raison d’être* on the right. Hix et al. ([12], p. 168) discovered this pattern as well, and proposed an additional explanation—these coalitions also relate to a form of government-opposition dynamic that is rooted at the national level, but is reflected in voting patterns at the European level.

In general, we observe two main differences between the *Alpha* and ERGM results: the baseline cooperation as estimated by *Alpha* is higher, and the ordering of coalitions from the strongest to the weakest is not exactly the same. The reason is the same as for the cohesion, namely different treatment of non-voting and abstaining MEPs. When they are ignored, as by *Alpha*, the baseline level of inter-group co-voting is higher. When non-attending and abstaining is treated as voting differently, as by ERGM, it is considerably more difficult to achieve co-voting coalitions, specially when there are on average 25% MEPs that do not attend or abstain. Groups with higher non-attendance rates, such as *GUE-NGL* (34%) and *NI* (40%) are less likely to form coalitions, and therefore have relatively lower ERGM coefficients (Fig 7) than *Alpha* scores (Fig 6).

#### Coalitions on Twitter

**Fig 8:**
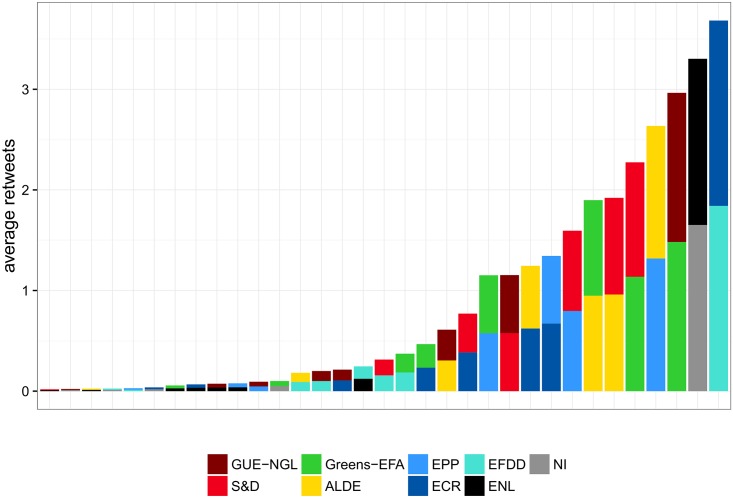
Coalitions between political groups as estimated by retweeting between members of different groups. The average number of retweets between different political groups is 0.8.

The first insight into coalition formation on Twitter can be observed in the retweet network in Fig 2B. The ideological left to right alignment of the political groups is reflected in the retweet network. Fig 8 shows the strength of the coalitions on Twitter, as estimated by the number of retweets between MEPs from different groups. The strongest coalitions are formed between the right-wing groups *EFDD* and *ECR*, as well as *ENL* and *NI*. At first, this might come as a surprise, since these groups do not form strong coalitions in the European Parliament, as can be seen in Figs 6 and 7. On the other hand, the MEPs from these groups are very active Twitter users. As previously stated, MEPs from *ENL* and *EFDD* post the largest number of retweets. Moreover, 63% of the retweets outside of *ENL* are retweets of *NI*. This effect is even more pronounced with MEPs from *EFDD*, whose retweets of *ECR* account for 74% of their retweets from other groups.

In addition to these strong coalitions on the right wing, we find coalition patterns to be very similar to the voting coalitions observed in the European Parliament, seen in Figs 6 and 7. The strongest coalitions, which come immediately after the right-wing coalitions, are between *Greens-EFA* on the one hand, and *GUE-NGL* and *S&D* on the other, as well as *ALDE* on the one hand, and *EPP* and *S&D* on the other. These results corroborate the role of *ALDE* and *Greens-EFA* as intermediaries in the European Parliament, not only in the legislative process, but also in the debate on social media.

#### Formation of coalitions

To better understand the formation of coalitions in the European Parliament and on Twitter, we examine the strongest cooperation between political groups at three different thresholds. For co-voting coalitions in the European Parliament we choose a high threshold of *Alpha* > 0.75, a medium threshold of *Alpha* > 0.25, and a negative threshold of *Alpha* < −0.25 (which corresponds to strong oppositions). In this way we observe the overall patterns of coalition and opposition formation in the European Parliament and in the two specific policy areas. For cooperation on Twitter, we choose a high threshold of $\bar{RT}>10$, a medium threshold of $\bar{RT}>1$, and a very low threshold of $\bar{RT}<0.001$.

**Fig 9:**
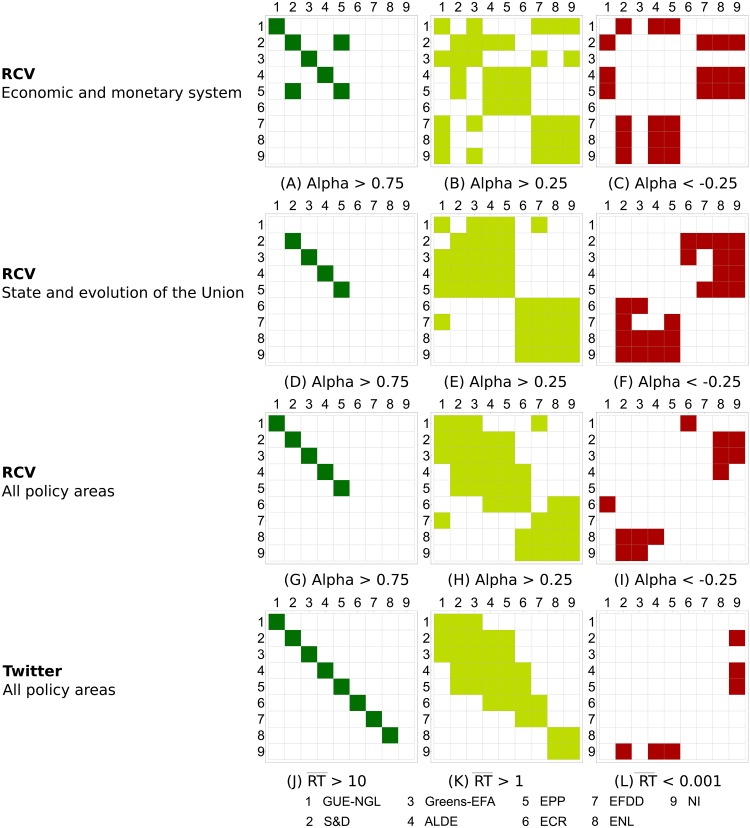
Formation of coalitions (*Alpha* > 0) and oppositions (*Alpha* < 0) in terms of the co-voting agreement (RCV). In the case of Twitter (the bottom charts), coalitions are indicated by higher average retweets ($\bar{RT}$), and oppositions by lower average retweets.

The strongest cooperations in the European Parliament over all policy areas are shown in Fig 9G. It comes as no surprise that the strongest cooperations are within the groups (in the diagonal). Moreover, we again observe *GUE-NGL*, *S&D*, *Greens-EFA*, *ALDE*, and *EPP* as the most cohesive groups. In Fig 9H, we observe coalitions forming along the diagonal, which represents the seating order in the European Parliament. Within this pattern, we observe four blocks of coalitions: on the left, between *GUE-NGL*, *S&D*, and *Greens-EFA*; in the center, between *S&D*, *Greens-EFA*, *ALDE*, and *EPP*; on the right-center between *ALDE*, *EPP*, and *ECR*; and finally, on the far-right between *ECR*, *EFDD*, *ENL*, and *NI*. Fig 9I shows the strongest opposition between groups that systematically disagree in voting. The strongest disagreements are between left- and right-aligned groups, but not between the left-most and right-most groups, in particular, between *GUE-NGL* and *ECR*, but also between *S&D* and *Greens-EFA* on one side, and *ENL* and *NI* on the other.

In the area of *Economic and monetary system* we see a strong cooperation between *EPP* and *S&D* (Fig 9A), which is on a par with the cohesion of the most cohesive groups (*GUE-NGL*, *S&D*, *Greens-EFA*, *ALDE*, and *EPP*), and is above the cohesion of the other groups. As pointed out in the section “Coalitions in the European Parliament”, there is a strong separation in two blocks between supporters and opponents of European integration, which is even more clearly observed in Fig 9B. On one hand, we observe cooperation between *S&D*, *ALDE*, *EPP*, and *ECR*, and on the other, cooperation between *GUE-NGL*, *Greens-EFA*, *EFDD*, *ENL*, and *NI*. This division in blocks is seen again in Fig 9C, which shows the strongest disagreements. Here, we observe two blocks composed of *S&D*, *EPP*, and *ALDE* on one hand, and *GUE-NGL*, *EFDD*, *ENL*, and *NI* on the other, which are in strong opposition to each other.

In the area of *State and Evolution of the Union* we again observe a strong division in two blocks (see Fig 9E). This is different to the *Economic and monetary system*, however, where we observe a far-left and far-right cooperation, where the division is along the traditional left-right axis.

The patterns of coalitions forming on Twitter closely resemble those in the European Parliament. In Fig 9J we see that the strongest degrees of cooperation on Twitter are within the groups. The only group with low cohesion is the *NI*, whose members have only seven retweets between them. The coalitions on Twitter follow the seating order in the European Parliament remarkably well (see Fig 9K). What is striking is that the same blocks form on the left, center, and on the center-right, both in the European Parliament and on Twitter. The largest difference between the coalitions in the European Parliament and on Twitter is on the far-right, where we observe *ENL* and *NI* as isolated blocks.

#### Relation between co-voting and retweeting

**Fig 10:**
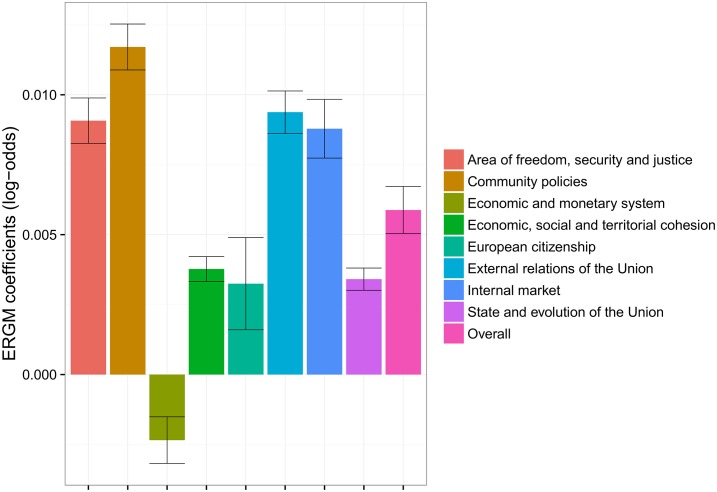
Influence of retweeting on the co-voting of MEPs as computed by ERGM. The influence is mostly positive, except for one policy area (*Economic and monetary system*), but very low. The ERGM coefficients of 0.01 correspond to an increase of probability from the chance of 0.5 to 0.503.

The results shown in Fig 10 quantify the extent to which communication in one social context (Twitter) can explain cooperation in another social context (co-voting in the European Parliament). A positive value indicates that the matching behavior in the retweet network is similar to the one in the co-voting network, specific for an individual policy area. On the other hand, a negative value implies a negative “correlation” between the retweeting and co-voting of MEPs in the two different contexts.

The bars in Fig 10 correspond to the coefficients from the edge covariate terms of the ERGM, describing the relationship between the retweeting and co-voting behavior of MEPs. The coefficients are aggregated for individual policy areas by means of a meta-analysis.

Overall, we observe a positive correlation between retweeting and co-voting, which is significantly different from zero. The strongest positive correlations are in the areas *Area of freedom, security and justice*, *External relations of the Union*, and *Internal markets*. Weaker, but still positive, correlations are observed in the areas *Economic, social and territorial cohesion*, *European citizenship*, and *State and evolution of the Union*. The only exception, with a significantly negative coefficient, is the area *Economic and monetary system*. This implies that in the area *Economic and monetary system* we observe a significant deviation from the usual co-voting patterns. Results from section “Coalitions in the European Parliament”, confirm that this is indeed the case. Especially noteworthy are the coalitions between *GUE-NGL* and *Greens-EFA* on the left wing, and *EFDD* and *ENL* on the right wing. In the section “Coalitions in the European Parliament” we interpret these results as a combination of Euroscepticism on both sides, motivated on the left by a skeptical attitude towards the market orientation of the EU, and on the right by a reluctance to give up national sovereignty.

## Discussion

We study cohesion and coalitions in the Eighth European Parliament by analyzing, on one hand, MEPs’ co-voting tendencies and, on the other, their retweeting behavior.

We reveal that the most cohesive political group in the European Parliament, when it comes to co-voting, is *Greens-EFA*, closely followed by *S&D* and *EPP*. This is consistent with what VoteWatch [38] reported for the Seventh European Parliament. The non-aligned (*NI*) come out as the least cohesive group, followed by the Eurosceptic *EFDD*. Hix and Noury [12] also report that nationalists and Eurosceptics form the least cohesive groups in the Sixth European Parliament. We reaffirm most of these results with both of the two employed methodologies. The only point where the two methodologies disagree is in the level of cohesion for the left-wing *GUE-NGL*, which is portrayed by ERGM as a much less cohesive group, due to their relatively lower attendance rate. The level of cohesion of the political groups is quite stable across different policy areas and similar conclusions apply.

On Twitter we can see results that are consistent with the RCV results for the left-to-center political spectrum. The exception, which clearly stands out, is the right-wing groups *ENL* and *EFDD* that seem to be the most cohesive ones. This is the direct opposite of what was observed in the RCV data. We speculate that this phenomenon can be attributed to the fact that European right-wing groups, on a European but also on a national level, rely to a large degree on social media to spread their narratives critical of European integration. We observed the same phenomenon recently during the Brexit campaign [39]. Along our interpretation the Brexit was “won” to some extent due to these social media activities, which are practically non-existent among the pro-EU political groups. The fact that *ENL* and *EFDD* are the least cohesive groups in the European Parliament can be attributed to their political focus. It seems more important for the group to agree on its anti-EU stance and to call for independence and sovereignty, and much less important to agree on other issues put forward in the parliament.

The basic pattern of coalition formation, with respect to co-voting, can already be seen in Fig 2A: the force-based layout almost completely corresponds to the seating order in the European Parliament (from the left- to the right-wing groups). A more thorough examination shows that the strongest cooperation can be observed, for both methodologies, between *EPP*, *S&D*, and *ALDE*, where *EPP* and *S&D* are the two largest groups, while the liberal *ALDE* plays the role of an intermediary in this context. On the other hand, the role of an intermediary between the far-left *GUE-NGL* and its center-left neighbor, *S&D*, is played by the *Greens-EFA*. These three parties also form a strong coalition in the European Parliament. On the far right of the spectrum, the non-aligned, *EFDD*, and *ENL* form another coalition. This behavior was also observed by Hix et al. [13], stating that alignments on the left and right have in recent years replaced the “Grand Coalition” between the two large blocks of Christian Conservatives (*EPP*) and Social Democrats (*S&D*) as the dominant form of finding majorities in the European Parliament. When looking at the policy area *Economic and monetary system*, we see the same coalitions. However, interestingly, *EFDD*, *ENL*, and *NI* often co-vote with the far-left *GUE-NGL*. This can be attributed to a certain degree of Euroscepticism on both sides: as a criticism of capitalism, on one hand, and as the main political agenda, on the other. This pattern was also discovered by Hix et al. [13], who argued that these coalitions emerge from a form of government-opposition dynamics, rooted at the national level, but also reflected at the European level.

When studying coalitions on Twitter, the strongest coalitions can be observed on the right of the spectrum (between *EFDD*, *ECR*, *ENL*, and *NI*). This is, yet again, in contrast to what was observed in the RCV data. The reason lies in the anti-EU messages they tend to collectively spread (retweet) across the network. This behavior forms strong retweet ties, not only within, but also between, these groups. For example, MEPs of *EFDD* mainly retweet MEPs from *ECR* (with the exception of MEPs from their own group). In contrast to these right-wing coalitions, we find the other coalitions to be consistent with what is observed in the RCV data. The strongest coalitions on the left-to-center part of the axis are those between *GUE-NGL*, *Greens-EFA*, and *S&D*, and between *S&D*, *ALDE*, and *EPP*. These results reaffirm the role of *Greens-EFA* and *ALDE* as intermediaries, not only in the European Parliament but also in the debates on social media.

Last, but not least, with the ERGM methodology we measure the extent to which the retweet network can explain the co-voting activities in the European Parliament. We compute this for each policy area separately and also over all RCVs. We conclude that the retweet network indeed matches the co-voting behavior, with the exception of one specific policy area. In the area *Economic and monetary system*, the links in the (overall) retweet network do not match the links in the co-voting network. Moreover, the negative coefficients imply a radically different formation of coalitions in the European Parliament. This is consistent with the results in Figs 6 and 7 (the left-hand panels), and is also observed in Fig 9 (the top charts). From these figures we see that in this particular case, the coalitions are also formed between the right-wing groups and the far-left GUE-NGL. As already explained, we attribute this to the degree of Euroscepticism that these groups share on this particular policy issue.

## Conclusions

In this paper we analyze (co-)voting patterns and social behavior of members of the European Parliament, as well as the interaction between these two systems. More precisely, we analyze a set of 2535 roll-call votes as well as the tweets and retweets of members of the MEPs in the period from October 2014 to February 2016. The results indicate a considerable level of correlation between these two complex systems. This is consistent with previous findings of Cherepnalkoski et al. [23], who reconstructed the adherence of MEPs to their respective political or national group solely from their retweeting behavior.

We employ two different methodologies to quantify the co-voting patterns: Krippendorff’s *Alpha* and ERGM. They were developed in different fields of research, use different techniques, and are based on different assumptions, but in general they yield consistent results. However, there are some differences which have consequences for the interpretation of the results.

*Alpha* is a measure of agreement, designed as a generalization of several specialized measures, that can compare different numbers of observations, in our case roll-call votes. It only considers yes/no votes. Absence and abstention by MEPs is ignored. Its baseline (*Alpha* = 0), i.e., co-voting by chance, is computed from the yes/no votes of all MEPs on all RCVs.

ERGMs are used in social-network analyses to determine factors influencing the edge formation process. In our case an edge between two MEPs is formed when they cast the same yes/no vote within a RCV. It is assumed that a priori each MEP can form a link with any other MEP. No assumptions about the presence or absence of individual MEPs in a voting session are made. Each RCV is analyzed as a separate binary network. The node set is thereby kept constant for each RCV network. While the ERGM departs from the originally observed network, where MEPs who didn’t vote or abstained appear as isolated nodes, links between these nodes are possible within the network sampling process which is part of the ERGM optimization process. The results of several RCVs are aggregated by means of the meta-analysis approach. The baseline (ERGM coefficients = 0), i.e., co-voting by chance, is computed from a large sample of randomly generated networks.

These two different baselines have to be taken into account when interpreting the results of *Alpha* and ERGM. In a typical voting session, 25% of the MEPs are missing or abstaining. When assessing cohesion of political groups, all *Alpha* values are well above the baseline, and the average *Alpha* = 0.7. The average ERGM cohesion coefficients, on the other hand, are around the baseline. The difference is even more pronounced for groups with higher non-attendance/abstention rates like *GUE-NGL* (34%) and *NI* (40%). When assessing strength of coalitions between pairs of groups, *Alpha* values are balanced around the baseline, while the ERGM coefficients are mostly negative. The ordering of coalitions from the strongest to the weakest is therefor different when groups with high non-attendance/abstention rates are involved.

The choice of the methodology to asses cohesion and coalitions is not obvious. Roll-call voting is used for decisions which demand a simple majority only (for a description of the political parties as well as the roll-call voting procedure the reader is referred to S1 Appendix). One might however argue that non-attendance/abstention corresponds to a no vote, or that absence is used strategically. Also, the importance of individual votes, i.e., how high on the agenda of a political group is the subject, affects their attendance, and consequently the perception of their cohesion and the potential to act as a reliable coalition partner.

## Supporting Information

S1 Appendix About the European Parliament. The appendix contains details about the historical and legal framework, political groups, roll-call voting procedures, and different policy areas that are the subject of roll-call votes.
(PDF)

## References

[1] Gayo-Avello D, Metaxas PT, Mustafaraj E. Limits of electoral predictions using Twitter. In: Proc. 5th Intl. AAAI Conf. on Weblogs and Social Media; 2011. p. 490–493.

[2] Eom YH, Puliga M, Smailović J, Mozetič I, Caldarelli G. Twitter-based analysis of the dynamics of collective attention to political parties. PLoS ONE. 2015;10(7):e0131184 Available from: 10.1371/journal.pone.0131184 26161795 PMC4498694

[3] Bollen J, Mao H, Zeng X. Twitter mood predicts the stock market. Journal of Computational Science. 2011;2(1):1–8. 10.1016/j.jocs.2010.12.007

[4] Ranco G, Aleksovski D, Caldarelli G, Grčar M, Mozetič I. The effects of Twitter sentiment on stock price returns. PLoS ONE. 2015;10(9):e0138441 Available from: 10.1371/journal.pone.0138441 26390434 PMC4577113

[5] Krippendorff K. Content Analysis, An Introduction to Its Methodology. 3rd ed Thousand Oaks, CA: Sage Publications; 2012.

[6] Hunter DR, Handcock MS, Butts CT, Goodreau SM, Morris M. ERGM: A package to fit, simulate and diagnose exponential-family models for networks. Journal of Statistical Software. 2008;24(3):1–29. 10.18637/jss.v024.i03 19756229 PMC2743438

[7] Attina F. The voting behaviour of the European Parliament members and the problem of the Europarties. European Journal of Political Research. 1990;18(5):557–579. 10.1111/j.1475-6765.1990.tb00248.x

[8] Kreppel A, Tsebelis G. Coalition formation in the European Parliament. Comparative Political Studies. 1999;32(8):933–966. 10.1177/0010414099032008002

[9] Quanjel M, Wolters M. Growing cohesion in the European Parliament. Annual Joint Sessions of the European Consortium for Political Research. 1993;.

[10] Brzinski JB. Political group cohesion in the European Parliament, 1989-1994. The state of the European Union. 1995;3:135–158.

[11] Hix S. Parliamentary behavior with two principals: Preferences, parties, and voting in the European Parliament. American Journal of Political Science. 2002;p. 688–698. 10.2307/3088408

[12] Hix S, Noury A. After enlargement: Voting patterns in the sixth European Parliament. Legislative Studies Quarterly. 2009;34(2):159–174. 10.3162/036298009788314282

[13] Hix S, Noury AG, Roland G. Democratic politics in the European Parliament. Cambridge University Press; 2007.

[14] Hix S, Noury A, Roland G. Power to the parties: Cohesion and competition in the European Parliament, 1979–2001. British Journal of Political Science. 2005;35(02):209–234. 10.1017/S0007123405000128

[15] Rice SA. Quantitative methods in politics. 1928;.

[16] Mozetič I, Grčar M, Smailović J. Multilingual Twitter sentiment classification: The role of human annotators. PLoS ONE. 2016;11(5):e0155036 Available from: 10.1371/journal.pone.0155036 27149621 PMC4858191

[17] Smailović J, Kranjc J, Grčar M, Žnidaršič M, Mozetič I. Monitoring the Twitter sentiment during the Bulgarian elections. In: Proc. IEEE Intl. Conf. on Data Science and Advanced Analytics; 2015. p. 1–10. Available from: 10.1109/DSAA.2015.7344886

[18] Conover M, Gonçalves B, Ratkiewicz J, Flammini A, Menczer F. Predicting the Political Alignment of Twitter Users. In: Proc. 3rd IEEE Conf. on Social Computing; 2011.

[19] Conover M, Ratkiewicz J, Francisco M, Gonçalves B, Flammini A, Menczer F. Political Polarization on Twitter. In: Proc. 5th Intl. AAAI Conf. on Weblogs and Social Media; 2011.

[20] Borondo J, Morales AJ, Losada JC, Benito RM. Characterizing and modeling an electoral campaign in the context of Twitter: 2011 Spanish Presidential election as a case study. Chaos. 2012;22(2). 10.1063/1.4729139 22757545

[21] Borondo J, Morales AJ, Benito RM, Losada JC. Mapping the online communication patterns of political conversations. Physica A: Statistical Mechanics and its Applications. 2014;414(0):403–413. 10.1016/j.physa.2014.06.089

[22] Larsson AO. The EU Parliament on Twitter—Assessing the Permanent Online Practices of Parliamentarians. Journal of Information Technology & Politics. 2014;0(0):1–18.

[23] Cherepnalkoski D, Mozetič I. Retweet networks of the European Parliament: Evaluation of the community structure. Applied Network Science. 2016;1:2 Available from: 10.1007/s41109-016-0001-4 30533494 PMC6245153

[24] Lazer D. Networks in Political Science: Back to the Future. PS: Political Science & Politics. 2011 1;44:61–68. 10.1017/S1049096510001873

[25] Porter MA, Mucha PJ, Newman MEJ, Warmbrand CM. A network analysis of committees in the U.S. House of Representatives. Proceedings of the National Academy of Science. 2005;102(20):7057–7062. 10.1073/pnas.0500191102 15897470 PMC1129104

[26] Zhang Y, Friend AJ, Traud AL, Porter MA, Fowler JH, Mucha PJ. Community structure in Congressional cosponsorship networks. Physica A: Statistical Mechanics and its Applications. 2008;387(7):1705–1712. 10.1016/j.physa.2007.11.004

[27] Waugh AS, Pei L, Fowler JH, Mucha PJ, Porter MA. Party Polarization in Congress: A Network Science Approach; 2009.

[28] Dal Maso C, Pompa G, Puliga M, Riotta G, Chessa A. Voting Behavior, Coalitions and Government Strength through a Complex Network Analysis. PLoS ONE. 2014;9(12):e116046 10.1371/journal.pone.0116046 25549351 PMC4280168

[29] Scherpereel JA, Wohlgemuth J, Schmelzinger M. The Adoption and Use of Twitter as a Representational Tool among Members of the European Parliament. European Politics and Society. 2016;p. 1–17. 10.1080/23745118.2016.1151125

[30] European Parliament. Roll-call votes data; 2016. [Online; accessed 29-January-2016]. Available from: http://www.europarl.europa.eu/RegistreWeb/search/typedoc.htm?codeTypeDocu=PPVD&leg=8

[31] European Parliament. Members of the EP; 2016. [Online; accessed 29-January-2016]. Available from: http://www.europarl.europa.eu/meps/en/xml.html?query=full&filter=all

[32] Cranmer SJ, Desmarais BA. Inferential network analysis with exponential random graph models. Political Analysis. 2011;19(1):66–86. 10.1093/pan/mpq037

[33] Robins G, Pattison P, Kalish Y, Lusher D. An introduction to exponential random graph (p*) models for social networks. Social Networks. 2007;29(2):173–191. Special Section: Advances in Exponential Random Graph (p*) Models. 10.1016/j.socnet.2006.08.002

[34] Lubbers MJ. Group composition and network structure in school classes: A multilevel application of the p* model. Social Networks. 2003;25(4):309–332. 10.1016/S0378-8733(03)00013-3

[35] Snijders TA, Baerveldt C. A multilevel network study of the effects of delinquent behavior on friendship evolution. Journal of Mathematical Sociology. 2003;27(2-3):123–151. 10.1080/00222500305892

[36] Krivitsky PN. Exponential-family random graph models for valued networks. Electronic Journal of Statistics. 2012;6:1100 10.1214/12-EJS696 24678374 PMC3964598

[37] Ripley RM, Snijders TA, Boda Z, Vörös A, Preciado P. Manual for RSIENA. University of Oxford, Department of Statistics, Nuffield College. 2011;1.

[38] VoteWatch. Voting in the 2009-2014 European Parliament: How do MEPs Vote after Lisbon; 2011. [Online; accessed 8-August-2016]. Available from: http://www.votewatch.eu/blog/wp-content/uploads/2011/01/votewatch_report_voting_behavior_26_january_beta.pdf

[39] Grčar M, Cherepnalkoski D, Mozetič I. The Hirsch index for Twitter: Influential proponents and opponents of Brexit. In: Proc. 5th Intl. Workshop on Complex Networks and their Applications. Studies in Computational Intelligence. Springer; 2016.

