# Supplementary material for: Cohesion and Coalition Formation in the European Parliament: Roll-Call Votes and Twitter Activities
Source: PLoS One. 2016 Nov 11;11(11):e0166586. doi: 10.1371/journal.pone.0166586 (PMC5106041; doi:10.1371/journal.pone.0166586)
Supplement: S1 Appendix — The appendix contains details about the historical and legal framework, political groups, roll-call voting procedures, and different policy areas that are the subject of roll-call votes. (PDF) [file pone.0166586.s001.pdf]

S1 Appendix

About the European Parliament

Historical and legal framework

The European Parliament is the only directly elected institution on the supranational level of the European Union. It occupies the legislative function in the political system of the EU. Since 1976 it is elected every 5 years by universal suffrage, which extends to every citizen of the EU, regardless of their country of residence. The parliament currently has 751 members, representing roughly 500 million citizens, corresponding to an electorate of roughly 375 million. The electorate is, in terms of size, second only to India. It is the largest supranational parliamentary body in the world [1].

Although the European Parliament for a long time had the reputation of being a toothless institution, its competences were successively extended in the recent years. The most important treaties in this respect were the Maastricht Treaty of 1993 and the Lisbon Treaty of 2009. The former granted the parliament veto rights for legislation coming from the council, putting it at eye level in the legislative procedure. The latter extended its competences to crucial policy areas, such as agriculture, immigration as well justice and internal affairs. Furthermore, the European Parliament now has the final word on the EU budget and has to approve the conclusion of international treaties, like trade agreements, in which the EU is a party [2, 3]. Since 2014 the parliament also has the right to nominate the head of the European Commission, which is considered to be the executive branch of the European political system [4].

The elections for the European Parliament are organized at the national level. Voters elect, depending on their country of residence, a national political group or list. The numbers of MEPs of each member state is roughly proportional to its population. Although larger countries in terms of population have more MEPs than smaller countries, the number of MEPs per inhabitant is higher for smaller countries. For example, while Germany, the most populous country elects 96 MEPs to the European Parliament, smaller countries like Slovenia or Luxembourg have 6 MEPs each. Along the definition of universal suffrage, European elections are therefore considered to be “free”, “direct” and “secret”; however, they are not considered “equal”. Since 2004 the electoral rules for European elections prescribe proportional representation for the elected MEPs [4, 5].

Political groups in the Eighth European Parliament

The voting data analyzed in this study corresponds to all 2535 roll-call votes conducted during the 8th European Parliament up to the end of February 2016. Currently, the parliament has 751 members. Most MEPs belong to one of the eight European political groups:

GUE-NGL: The European United Left–Nordic Green Left unite 52 radical left wing, socialist and communist parties from 14 European member states. Being generally integration friendly, the GUE-NGL wants to influence the integration process in the direction of a social union with the stronger protection of workers and a higher degree of wealth redistribution [6]. The Greek Syriza party, the Irish Sinn Féin, the German Die Linke and the French Front de gauche (of which the French Communist Party, PCF is a stake holder) are part of this group.

S&D: The Socialists & Democrats is second largest group in the European Parliament with 189 seats. It consists of social democratic, socialist and labour parties from most European member states. In the literature it is described as having a balanced stance between workers protection and market economy, and to be integration friendly [6]. This group, for example, contains the French PS (Parti socialiste), the German SPD (Sozialdemokratische Partei Deutschlands), and, for the time being, the British Labour Party.

Greens-EFA: The Group of the Greens and European Free Alliance is the political home to green (The Greens) and regionalist/separatist political parties (EFA) from 22 member states. At the moment, it holds 50 seats in the European Parliament. Being considered as favorable to European integration, this group strives for a more ecological and social union [6]. Apart from various green parties from all over Europe, the Greens-EFA group also summons different political movements with a separatist agenda. Better known national parties of this group are the Scottish National Party, the Valencian National Bloc, the Republican Left of Catalonia, and Basque Solidarity (all Spain).

ALDE: The Alliance of Liberals and Democrats for Europe summons liberal political groups from 20 European countries. Together with the EPP and the S&D it holds the majority in the European Parliament. It occupies 69 seats and is thus the fourth-largest group in the European Parliament. The literature describes them as cooperation and integration friendly, with a stance against overregulation and bureaucratization [6].

EPP: The European People's Party is the strongest group in the European Parliament with 216 seats. This group unites christian democrats, conservatives and center-right parties from most European member states. Hix et. al [6] describe them as "integration-friendly and [...] strong supporter[s] of the European Constitution". The ruling German CDU/CSU (Christian Democratic Union / Christian Social Union) and the French Les Reṕublicains (former UMP, under former president Nicolas Sarkozy) are parts of the EPP.

ECR: European Conservatives and Reformists hold 73 seats and are the third largest group in the parliament. Originally being a split-off from the EPP over an argument about the Lisbon treaty, it provides a political home

to conservative and Eurosceptic parties from 18 EU member states [7]. The ruling Polish Law and Justice Party (PiS) and the British Conservative Party are parts of the ECR.

EFDD: The Europe of Freedom and Direct Democracy holds 46 seats, uniting extreme right-wing and Eurosceptic parties from eight countries [6, 7]. Part of this group are, for example, the Italian Five Star Movement (Movimento 5 Stelle), the Sweden Democrats, and the UK Independence Party (UKIP).

ENL: The Europe of Nations and Freedom is the smallest group in the European Parliament with 39 seats. Its extreme right-wing member organizations come from nine different countries. The biggest are the French Front National, the Italian Lega Nord, and the Austrian Freedom Party (FPÖ).

NI: Apart from the eight groups described above, there are currently 16 non-aligned (Non-Inscrits NI) members in the European Parliament. These MEPs are partly split-offs from national lists organized in one of the above groups (like Bruno Gollnisch from the FN), or are part of other minoritarian extreme-right wing political movements, like the Hungarian Jobbik, the German National Democratic Party (NPD), or the Greek Golden Dawn (XA). In our analysis we treat the group of non-aligned MEPs as one political group.

In the European Parliament a simple majority corresponds to 376 votes. A coalition between the two major groups, EPP and S&D could theoretically ensure 404 votes. Coalitions on the left with S&D, Greens-EFA and GUE-NGL (all together 291 seats), or on the right with EPP, ECR, EFDD and ENL (all together 374 seats) would, however, stay below the 376 vote margin. The majority ratios leave the liberal ALDE with the role of natural intermediary between both sides of the political spectrum. In general, especially taking into account the ideological provenience of most MEPs in the NI group, the majority in the European Parliament consists of the right-side of the political spectrum.

Roll-call voting in the European Parliament

Following parliamentary traditions, voting in the EP is mainly carried out via a show of hands. The president of the session then decides by visual judgment whether the majority is for or against the proposal in question. This method represents a fast and simple way to determine majorities in votes were only a simple majority is necessary. In certain cases, however, the votes are undertaken using roll-call votes [8]:

1. For tight decision or if MEPs believe the president's judgment is wrong, the decision can be contested. The president then has the possibility to decide to hold the vote again, this time using the electronic voting system (Rule 181).

2. Roll-call votes are used at the request of at least 40 members of the parliament, at the latest on the evening prior to the parliamentary session (Rule 180). A request for a secret vote formed by the same number, however, overrules the roll-call vote request.

3. The election of the European Commission (Rule 118). A motion of no confidence in the members of the European Commission. In reference to rule 180, if the vote is based on a report and concerns an amendment thereof (Rules 173, 179).

4. The final decision of the budgetary conciliation process is taken via a roll-call vote (Rule 90).

5. In committees of the European Parliament if requested so by at least one quarter of its members.

Roll-call voting in the historical sense comprises calling each individual EP member alphabetically by name, who then expresses a “yes”, “no”, or “abstain” with regard to the vote in question. While the procedural rules of the European Parliament still foresee this oral approach of establishing majorities (Rule 180), most of the roll-call votes today are held using electronic voting. Therefore, MEPs are required to identify themselves using an electronic identification card. In any case, the results of roll-call votes, indicating how each individual MEP who was present had voted (Rule 181), are recorded in the minutes of the parliamentary session. Data from this session’s minutes form the basis of our analysis.

Policy areas

The roll-call voting data covers eight policy areas. In what follows, a short description of the different policy areas is given [9, 10]:

Area of freedom, security and justice: This policy area concerns home affairs and policies with regard to justice and the freedom of movement within the EU. It also covers the harmonization of legal systems and cooperation at the level of police and justice between Member States. Well known projects attributed to this policy area are the Schengen Area, the European Arrest Warrant and Frontex patrols.

Community policies: Concerns European political parties and the European Parliament’s interaction with other institutions, on the European and national levels. It also covers the implementation of European treaties and all general issues with regard to the EU institutions.

Economic and monetary system: This policy area covers all matters concerning the economic and monetary system of the EU and regulations with regard to financial regulations, taxation, competition, and the free movement of capital and payments. It also comprises the relationship to the European Central Bank (which is accountable to the European Parliament).

Economic, social and territorial cohesion: Questions with regard to employment and social, but also infrastructural policies, which should promote the economic, social and territorial cohesion of the EU are covered by this policy area.

European citizenship: Concerns matters of citizenship, migration and asylum.

External relations of the Union: Covers policies regulating the relationship of the EU with third countries. Decisions concerning the accession process to the EU, associations, and the European neighbourhood policy fall in this category. Security, defence, development policy, and human-rights matters are in also this policy area.

Internal market: This policy area deals with internal market regulations, competition and consumer rights. It also covers standards for products, public subsidies and the mutual recognition of degrees and qualifications.

State and evolution of the Union: This policy area covers seminal decisions with regard to the European integration process and concerns the power balance between the EU institutions on the one side and the Member States on the other side.

References

[1] European Parliament. About the European Parliament. 2016;Available from: http://www.europarl.europa.eu/us/en/about_eu_parliament.html.

[2] Hix S, Høyland B. Empowerment of the European parliament. Annual Review of Political Science. 2013;16:171–189.

[3] European Parliament. Development of the Parliament's Legislative Role. 2016;Available from: http://www.europarl.europa.eu/us/en/about_eu_parliament/parliament_info_articles/legislative_development.htmlhttp;jsessionid=73FE06549FDAC456DA707C5E838641C1.

[4] European Parliament. European Elections. 2016;Available from: http://www.europarl.europa.eu/atyourservice/en/20150201PVL00036/Elections.

[5] European Parliament. Composition, Powers and Functions. 2016;Available from: http://www.europarl.europa.eu/us/en/about_eu_parliament/parliament_info_articles/composition_power_functions.htmlhttp;jsessionid=3A0C6FEF7497450AC7C97DF9C4AF1746.

[6] Hix S, Noury A. After enlargement: voting patterns in the sixth European Parliament. Legislative Studies Quarterly. 2009;34(2):159–174.

[7] Slomp H. Europe, a Political Profile: An American Companion to European Politics. vol. 1. ABC-CLIO; 2011.

[8] European Parliament. Roles of Procedure; 2015. 8th parliamentary term. Available from: http://www.europarl.europa.eu/sides/getDoc.do?pubRef=-//EP//NONSGML+RULES-EP+20150909+0+DOC+PDF+V0//EN&language=EN.

[9] Meserve SA, Pemstein D, Bernhard WT. Political ambition and legislative behavior in the European Parliament. The Journal of Politics. 2009;71(03):1015–1032.

[10] European Parliament. Topic Areas; 2016. [Online; accessed 29-July-2016]. Available from: `http://www.europarl.europa.eu/news/en/news-room/topic/`.
